# Ixodid ticks of Western Palearctic bats: ecology, host-parasite relationships, geographic distribution and zoonotic importance

**DOI:** 10.3389/fvets.2025.1517704

**Published:** 2025-06-18

**Authors:** Attila D. Sándor, Cristian Domșa, Áron Péter, Sándor Hornok

**Affiliations:** ^1^HUN-REN-UVMB Climate Change: New Blood-Sucking Parasites and Vector-Borne Pathogens Research Group, Budapest, Hungary; ^2^Department of Parasitology and Zoology, University of Veterinary Medicine, Budapest, Hungary; ^3^STAR-UBB Institute, Babes-Bolyai University, Cluj-Napoca, Romania; ^4^Romanian Ornithological Society, Cluj Napoca, Romania

**Keywords:** Chiroptera, host-specificity, Ixodidae, vector-borne pathogens, zoonotic diseases

## Abstract

Bats in the Western Palearctic are host for diverse array of ectoparasites, including three ixodid ticks (*Ixodes ariadnae*, *I. simplex*, and *I. vespertilionis*), which are highly specialized to parasitize these mammals. In this study we collected and analyzed 3,965 host-tick records across 31 bat species from published literature, online sources, and unpublished field data. Individual bat-specialist ticks showed distinct host preferences, with cave-dwelling bats accounting for over 90% of all records. *Ixodes vespertilionis* was the most generalist of them, with a broad host range and distribution, while *I. simplex* was highly host-specific, primarily parasitizing a single host species, *Miniopterus schreibersii*. *Ixodes ariadnae* had a similar host spectrum as *I. vespertilionis* but more restricted geographical range, likely influenced by seasonal and life history factors. Our findings revealed substantial geographical overlap in tick distributions across Central and Eastern Europe. Free-living tick stages were predominantly found in caves, and males were observed more frequently than females. Non-bat specific, as well generalist ticks such as *Ixodes ricinus* and *Rhipicephalus sanguineus* s.l. were rare on bats, with larger bat species being the more common hosts. These ticks may host DNA of several bacterial, viral, and parasitic pathogens, suggesting an important role in pathogen transmission to bats and possibly other mammals. This study underscores the ecological significance of bat-specialist ticks and highlights the need for further research on their distribution, host interactions, and role in zoonotic disease transmission.

## Background

Ixodid ticks (Acari: Ixodidae) are obligate parasites of vertebrates, widely distributed across all terrestrial biomes of Earth (1). They are an ancient group, showing long coevolution with vertebrates, initially being the parasites of feathered dinosaurs/birds (2), later evolving to infest all terrestrial vertebrate groups (3). Currently, there are over 700 valid species, with high diversity in the tropics (4). Most species are specialized to feed either on birds, mammals or reptiles, however, several species are generalists, capable of feeding on most available terrestrial vertebrates in their habitats. In contrast, some species exhibit strict host specificity, adapting to feed on a single or very few host species (5, 6). Most ixodid ticks use two or three different hosts throughout their life cycle, with each developmental stage taking a single blood meal (with the exception of males). They attach to the hosts skin, penetrate it using their hypostome and chelicerae, then extract blood from the host, through a process called engorgement. Fully engorged ticks detach from the host in specific areas, they molt into the next development stage (larva to nymph to adult) or lay eggs (females) and die. Throughout this process, ticks may transmit pathogens (viral, bacterial or protozoan) between hosts, playing a crucial role in the epidemiology of vector-borne diseases (7, 8). Ticks are likely the most important vectors of pathogens in the temperate regions and show constant adaptation to changing climatic and biotic conditions, thus being in the forefront of zoonotic disease emergence (9).

Bats are among the most widespread terrestrial mammals, with high mobility and species diversity and they are important ecosystem service providers, too (8). They also may serve as important reservoir hosts for a wide range of pathogens, including viruses, bacteria, and parasites, some of which have the potential to spill over into livestock or human populations and cause emerging infectious diseases (10).

Recent studies of bat associated ectoparasites showed that these may carry DNA of a diverse array of viral, bacterial or protozoan pathogens, some with proven zoonotic character (11), although most remain uncharacterized (12–15). Among these, DNA of several pathogenic bacteria was identified in bat specialist ticks in Europe, Africa but also in the New World (16). In addition, ixodid ticks of Palearctic bats were suggested to play a role in the cycles of several groups of protozoa (17) and viruses (18). Two of the three bat specialist ticks occurring in the Western Palearctic are known to attack humans as well (19, 20). Furthermore, research on bat ticks is important from a taxonomic point of view, as reflected by the descriptions of six new bat-specialist ticks from Europe and Asia during the last decade (21–25), while current assessments are neglected in the region (26). In conclusion, the knowledge of bat-tick relations may provide valuable insights into the mechanisms driving host–parasite interactions and the importance of bat and tick populations in the ecology and spatial evolution of pathogens they may harbor. Here we intend to construct a general spatial distribution of hard ticks hosted by bats in the Western Palearctic, using georeferenced occurrences (mostly published in literature, but also from databases and some unpublished, own records) of specialist and generalist ixodid ticks registered on bats (or in case of bat specialist ticks in bat roosts). In addition, we intend to characterize the role of both the host-, as well the tick ecology may play in building these relations, with a special focus on their role in vector-borne pathogen spread.

## Methods

### Database creation

Our methodology followed a three-step process. First, a keyword search was performed using terms as: ‘ticks’ or ‘Ixodidae’ + ‘bats’, + ‘Western Palearctic’, or ‘*Ixodes ariadnae*’/‘*Ixodes simplex*’ and ‘*Ixodes vespertilionis*’ + ‘Western Palearctic’ in the following literature databases: PUBMED, Web of Science and Google Scholar. In the next step, duplicates were eliminated, and abstracts were verified to contain relevant data. This process resulted in a database of suitable papers. Subsequently, copies of the original publications were obtained and the references cited in these works were traced. This process was repeated until no new references were found. In the third step we extracted each individual host-tick record from the references, noting the location, date, host and parasite species, development stage (for ticks) and pathogen (if) mentioned. To complete the collated records, we traced museum specimen collections and observation records using data repositories like Global Biodiversity Information Facility,^1^ Obervation.org and NBN Atlas,^2^ among others. Direct internet searches using the same keywords also provided hits, verified by photos of the tick species. Unpublished data from our field studies in Algeria, Bulgaria, Hungary, and Romania (2019–2023) were also included. These records were introduced into a database and individually georeferenced to create distribution maps.

### Distribution maps

For the maps, we overlaid the range of each host species with the presence data for each tick species. Each host range was set with transparency, so the more ranges overlapped, the more intense the range color appeared—a proxy for multiple host species presence. For the primary bat host species, we used freely available shapefiles from the International Union for Conservation of Nature (IUCN) Red List (27). IUCN ranges were used previously primarily for conservation biology of bats (28) or other mammals (29), but also for establishing the relationships between bats and argasid soft ticks (30), as well for bats’ insect ectoparasites and vectored pathogens (31). In the next step, we intersected these ranges with the contour of the Western Palearctic, which was delimited according to previously published borders (30, 32, 33).

### Host–parasite relationships

Using the database, we mapped each host–parasite relationship and classified hosts as primary or accidental. To determine primary or accidental hosts of any ixodid tick species, we applied an arbitrary rule: any bat species with more than 5.0% of the records for a particular tick species was considered a primary host. Hosts with fewer than 5.0% of cumulative records for a particular tick species were considered non-primary or accidental hosts, following a system previously proposed for bat-fly associations (34–36). Additional host-related information, such as roosting sites or reproductive stages, was also extracted from the primary publications where available. Hosts were assigned either into cave-dwelling, or crevice dwelling group, based on their roosting preferences in their active period (37).

## Results

In total, 507 published references were included in the primary reference database (Supplementary Table S1), of which 317 contained records of bat ticks. Additionally, 27 records were extracted from online sources, supplemented by 207 unpublished host–parasite records from the authors. The bat host—tick reference database contains 3,965 individual entries (Supplementary Table S1), with the three bat-specialist ticks making up to 97.3% of the total (Table 1), while ticks with generalist host selection were recorded on bats in 110 instances (Supplementary Table S1). The complete database contains 3,855 entries of bat specialist ticks (8,997 individual ticks), collected from 3,162 individual bat hosts (5,680 ticks), together with a total of 730 instances of ticks collected from the environment (3,225 individuals of unengorged, free ticks, collected generally from underground roosts’ walls), while collection circumstances were unknown for 92 cases (*n* = 92 ticks, only tick species and geographic location were recorded). Altogether 31 bat species were recorded to host bat-specialist ticks, with most records noted for *I. vespertilionis* (Table 1). For 24 cases, records mentioned only generic ‘Chiroptera,’ while 10 cases were assigned to either *Myotis* spp., *Pipistrellus* spp., or *Plecotus* spp. Only 2 cases (0.005% of all records) involved bat ticks found on non-bat hosts—both on humans. Genetic analysis of previous blood meals identified nine cases of non-bat hosts across two tick species (all host species are listed in Table 2).

**Table 1:** Bat-specialist ticks recorded in the Western Palearctic.

Tick species	Free stages	Collected from host	Total number of host species	Number of primary host species	Number of non-primary hosts	Non-bat host species	Unknown/Undefined host	Total
*Ixodes ariadnae*	25	107	15	6	9	0	0	132
*Ixodes simplex*	663	3,149	14	1	13	2	4	3,816
*Ixodes vespertilionis*	2,546	2,323	30	5	25	4	88	4,957
Total	3,234	5,579	31			4	92	8,905

Number of records with known hosts, free stages and host-types.

**Table 2:** Primary and non-primary bat host species of hard ticks (Ixodidae) in the Western Palearctic.

Tick species	Primary host species	Non-primary host species	Non-bat hosts
*Ixodes ariadnae*	*Myotis alcathoe* *Myotis bechsteinii* *Myotis daubentonii* *Myotis emarginatus* *Myotis myotis* *Plecotus auritus*	*Barbastella barbastellus* *Myotis blythii* *Myotis brandtii* *Myotis dasycneme* *Myotis nattereri* *Pipistrellus pygmaeus* *Rhinolophus ferrumequinum,* *Rhinolophus hipposideros* *Rhinolophus mehelyi*	–
*Ixodes simplex*	*Miniopterus schreibersii*	*Myotis alcathoe* *Myotis bechsteinii* *Myotis blythii* *Myotis daubentonii* *Myotis emarginatus* *Myotis myotis* *Myotis nattereri* *Nyctalus leisleri* *Pipistrellus kuhlii* *Rhinolophus euryale* *Rhinolophus ferrumequinum* *Rhinolophus hipposideros* *Rhinolophus mehelyi*	*Homo sapiens* *Canis lupus familiaris*
*Ixodes vespertilionis*	*Myotis myotis* *Myotis punicus* *Rhinolophus euryale* *Rhinolophus ferrumequinum* *Rhinolophus hipposideros*	*Asellia tridens* *Barbastella barbastellus* *Eptesicus serotinus* *Miniopterus schreibersii* *Myotis alcathoe* *Myotis bechsteinii* *Myotis blythii* *Myotis brandtii* *Myotis capaccinii* *Myotis dasycneme* *Myotis daubentonii* *Myotis emarginatus* *Myotis mystacinus* *Myotis nattereri* *Nyctalus noctula* *Pipistrellus kuhlii* *Pipistrellus pygmaeus* *Pipistrellus nathusii* *Pipistrellus pipistrellus* *Plecotus auritus* *Plecotus austriacus* *Rhinolophus blasii* *Rhinolophus mehelyi* *Rhinopoma muscatellum* *Vespertilio murinus*	*Homo sapiens* *Canis lupus familiaris* *Equus caballus* *Sus scrofa*

*Ixodes vespertilionis* had the most diverse host spectrum, with 30 different host species (5 primary and 25 non-primary hosts). *Ixodes ariadnae* had the most primary hosts (6), while *I. simplex* had a single primary host harboring 98.43% of all records. Most ticks were recorded on cave-dwelling bat species (93.6%), with a single species (*I. ariadnae*) occurring regularly on crevice-dwelling bat hosts (these include species which rely on rock-crevices, but also tree-hole roosting ones).

**Figure 1:**
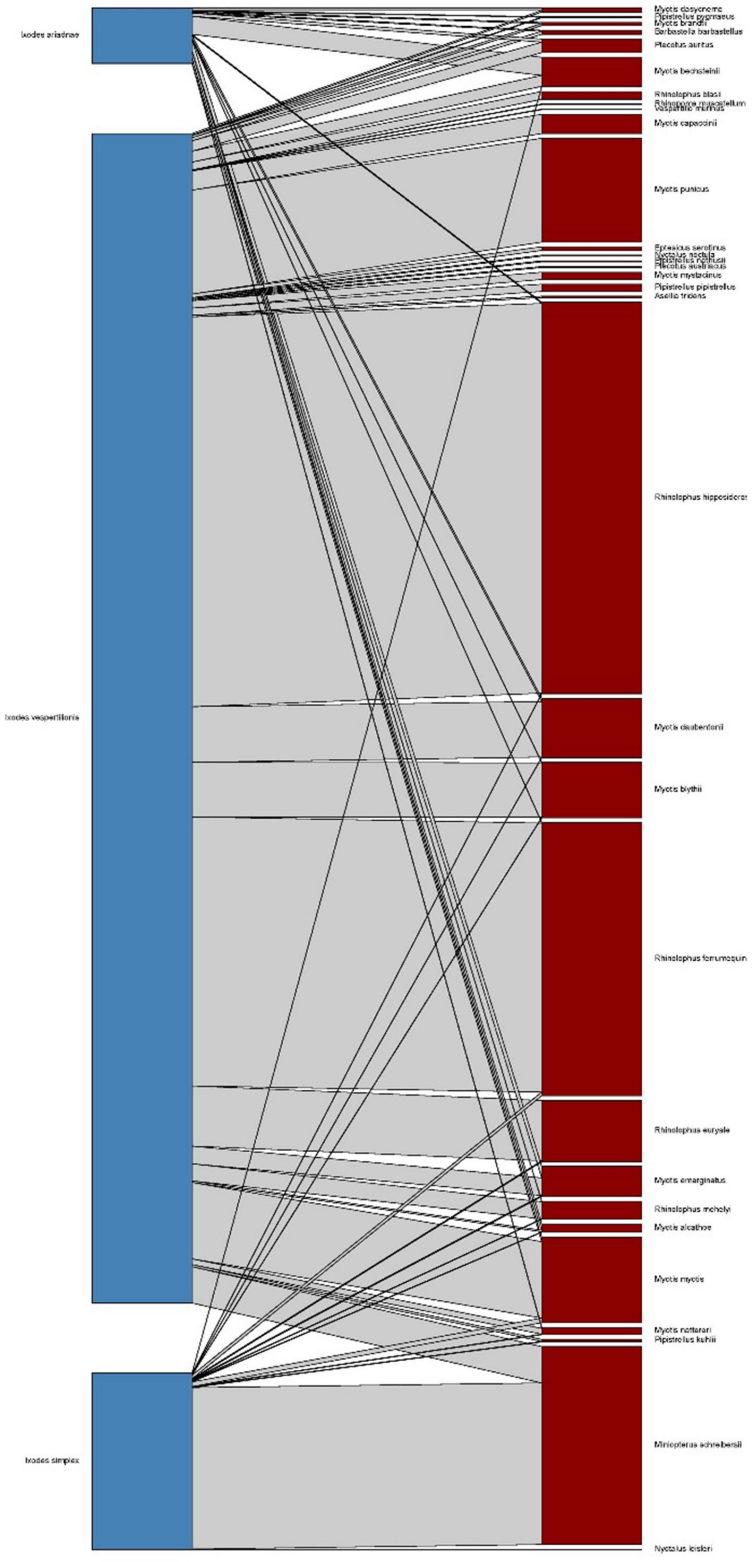
Bipartite representation of the parasite network of bats and their specific hard ticks using a quantitative interaction web based on individual host–parasite relations. Links between nodes represent the sum of individual bat tick occurrences for a given bat and tick species couple (*blue* bars – bat species, *dark red* bars – tick species, *grey* bars – host-parasite links).

Host-collected ticks were mainly subadult stages (90.2%), together with 537 adult females (9.7%) and 10 males collected from hosts (only in case of *I. vespertilionis* were males found on bats). The different tick species showed distinct host selection, with small overlap in host palette, mainly among hosts of *I. ariadnae* and *I. vespertilionis* (Figure 1). Free-stages of bat-specialist ticks were represented mainly by adults and were dominated by *I. vespertilionis* (675 individual records of 2,547 ticks, 78.7% of all free ticks), with a highly biased sex ratio toward males (1–2.32). Records of free individuals for the other two species are rare or accidental (Table 1). *Ixodes simplex* showed highly aggregated off-host presence (a single visit to a site used as nursery colony by *M. schreibersii* in the previous year resulted in 554 ticks collected from a crevice with an area of ca. 0.02 sqm, involving all tick developmental stages and sexes), but altogether only 23 instances of free individuals are known.

**Figure 2:**
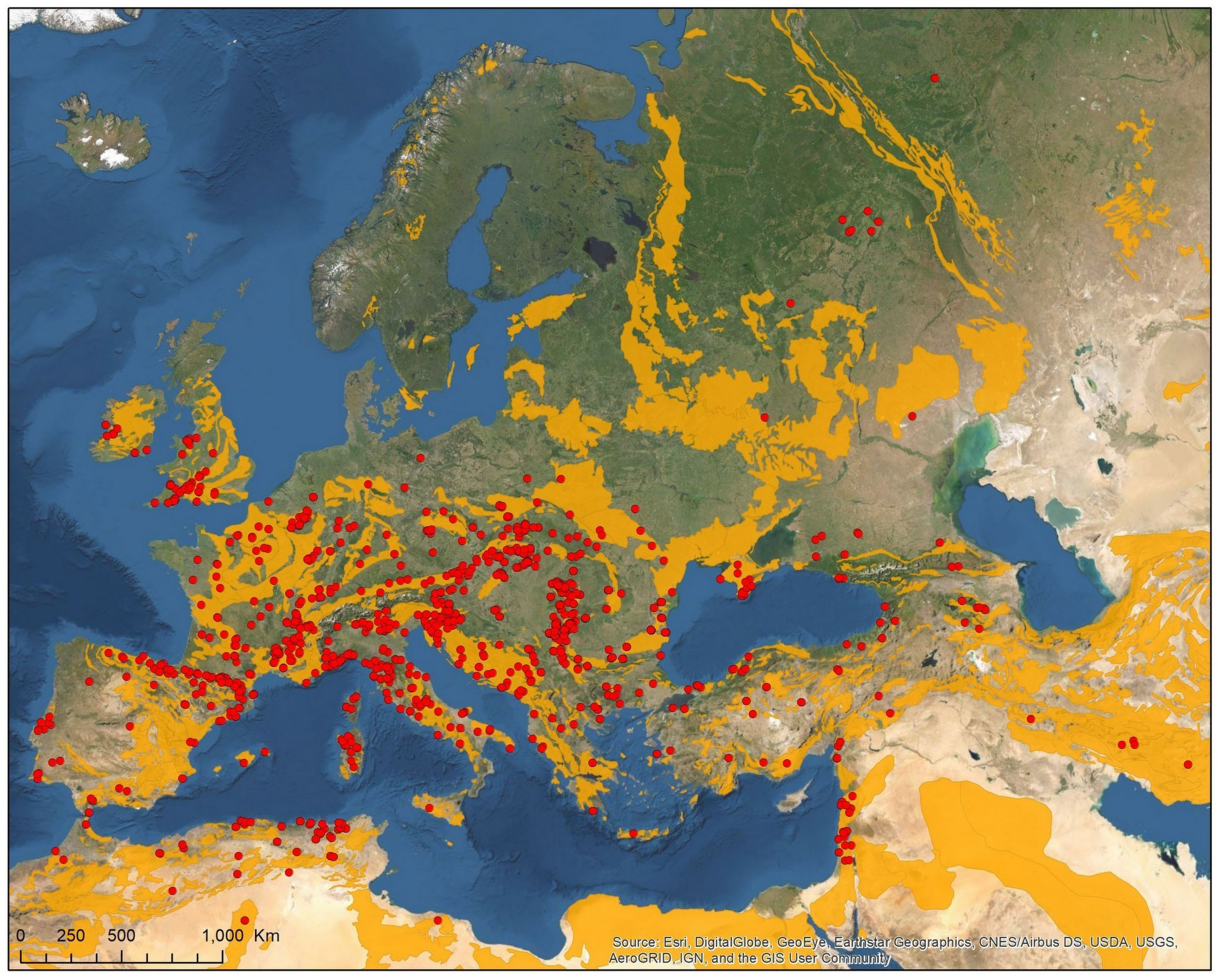
Geographic distribution of host-specialist bat ticks in the Western Palearctic (lime stone bedrock in *yellow*).

**Figure 3:**
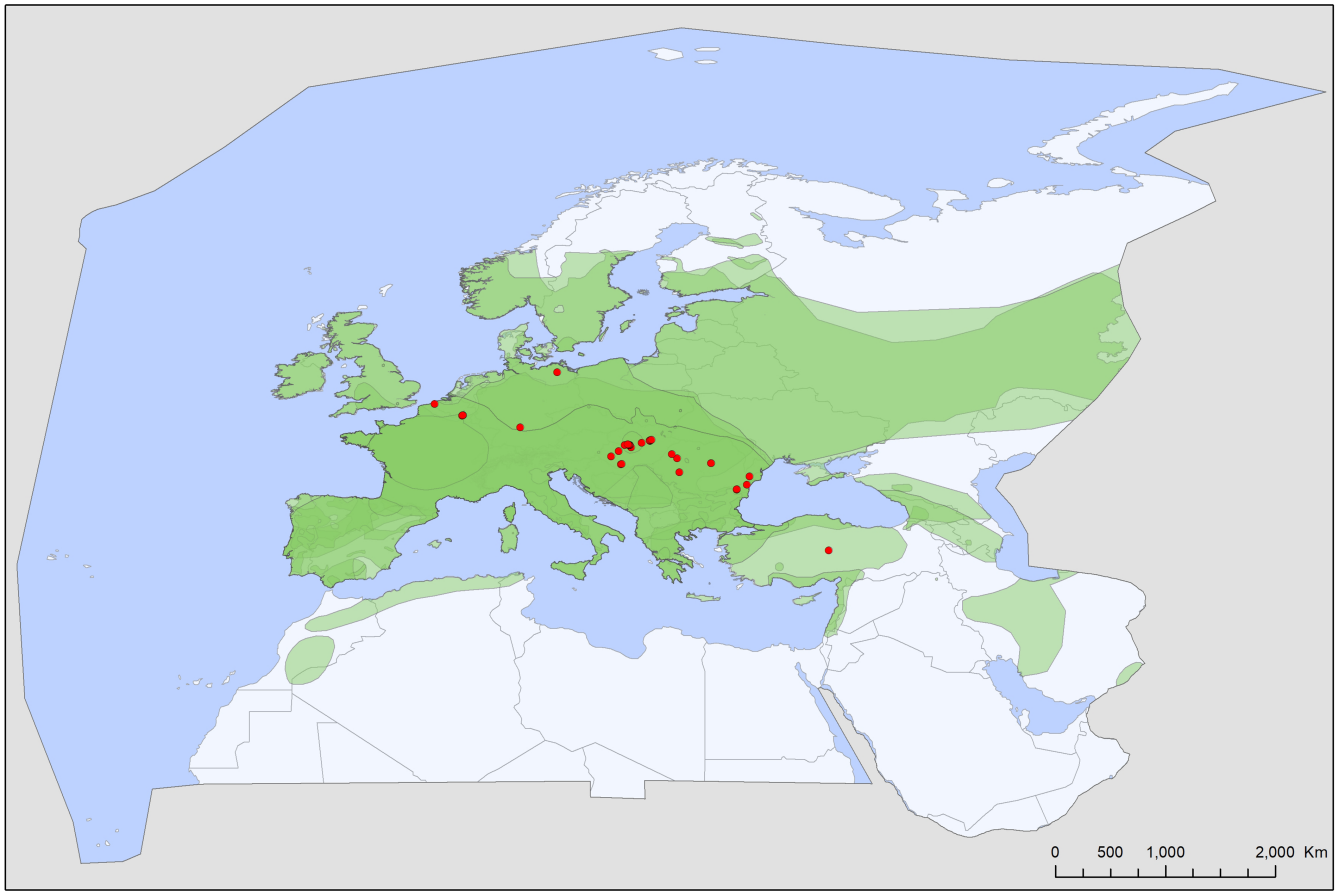
Geographic distribution of *Ixodes* *ariadnae* records in the Western Palearctic, overlaid to the geographic ranges for the six bat species studied as primary hosts (*Myotis alcathoe*, *M. bechsteinii*, *M. daubentonii*, *M. emarginatus*, *M. myotis*, *Plecotus auritus*) of this tick. Transparent layers were mapped on top of one another to highlight regions with dense range overlap.

**Figure 4:**
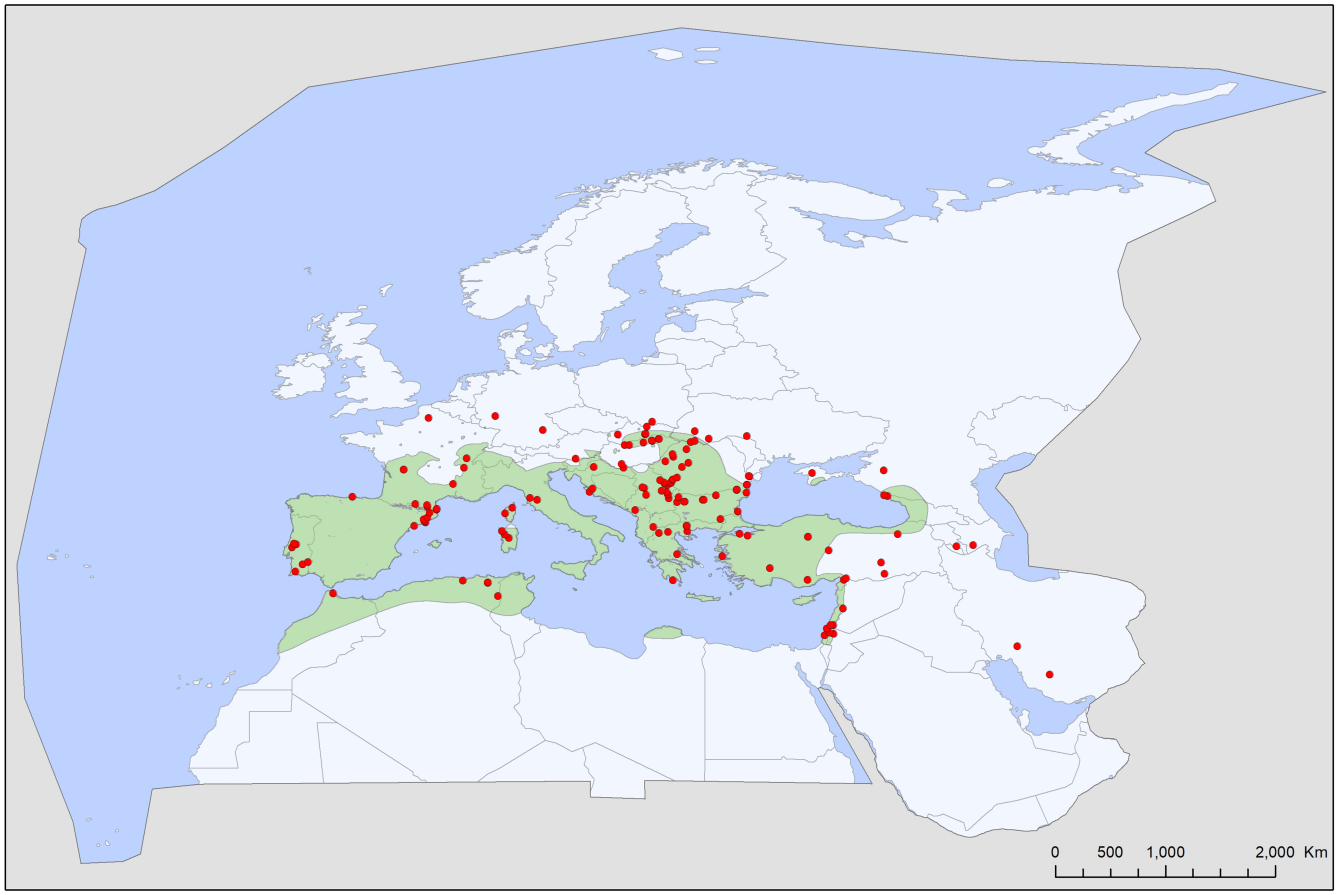
Geographic distribution of *Ixodes simplex* records in the Western Palearctic, overlaid to the geographic range for its primary host species, *Miniopterus schreibersii*.

**Figure 5:**
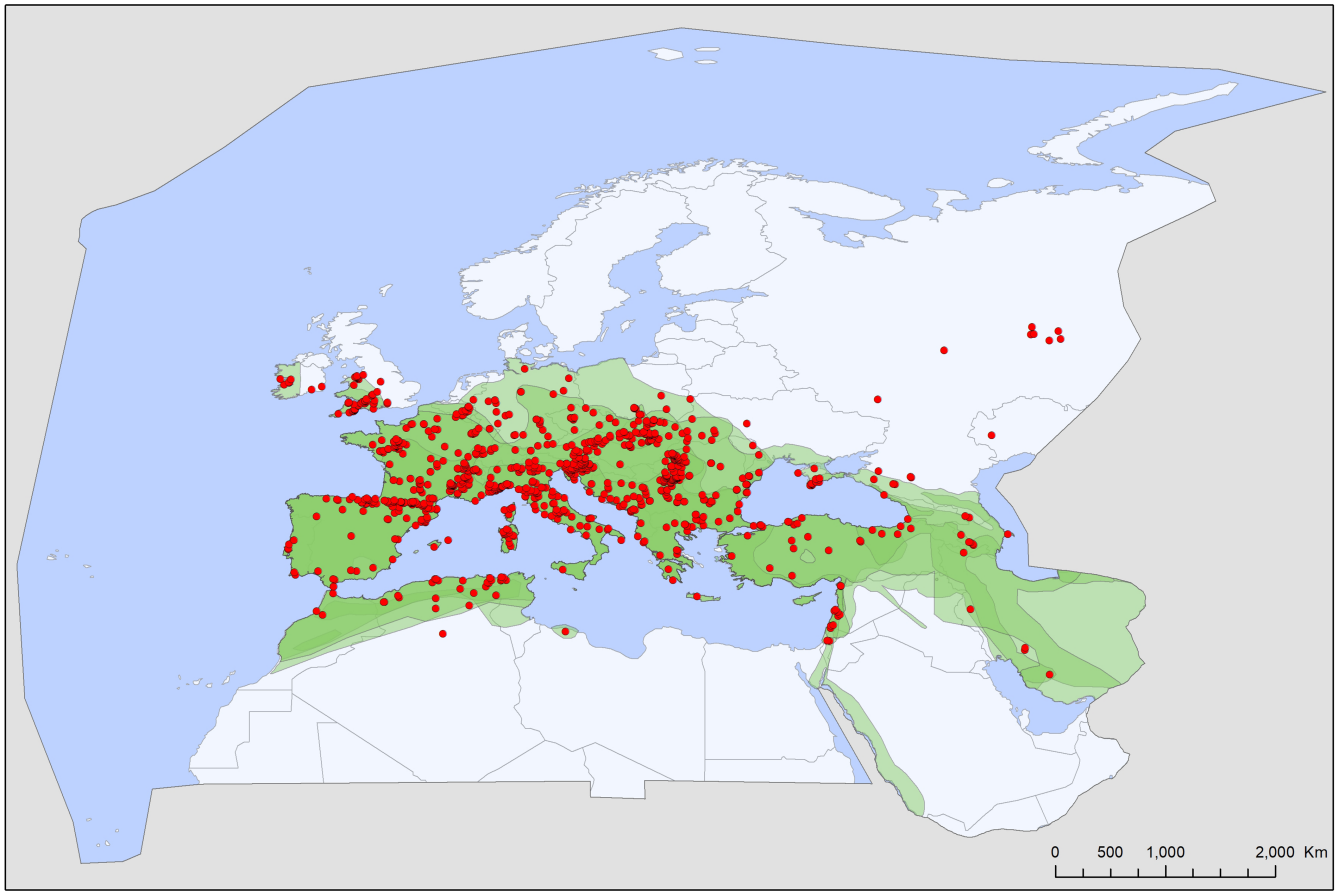
Geographic distribution of *Ixodes* *vespertilionis* records in the Western Palearctic, overlaid to the geographic ranges for the five bat species studied as primary hosts (*Myotis myotis*, *M. punicus*, *Rhinolophus euryale*, *R. ferrumequinum*, *R. hipposideros*) of this tick. Transparent layers were mapped on top of one another to highlight regions with dense range overlap. Some host species have additional range overlap in Africa and Central and South Asia.

**Figure 6:**
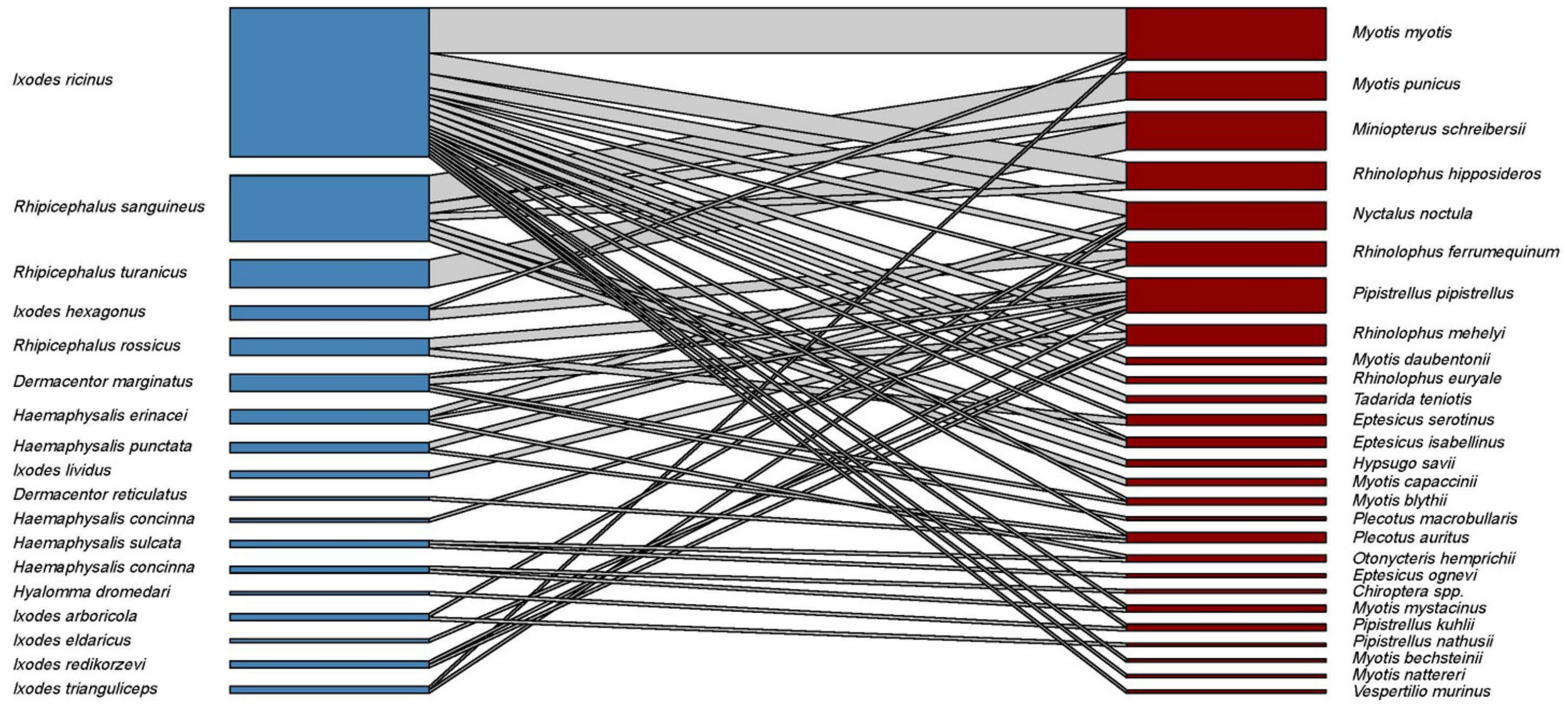
Bipartite representation of the parasite network of generalist/non-specialist ticks and their bat hosts using a quantitative interaction web based on individual host–parasite relations. Links between nodes represent the sum of individual bat tick occurrences for a given bat and tick species couple (*blue* bars – bat species, *dark red* bars – tick species, *grey* bars – host-parasite links).

Tick records showed wide geographic distribution, with range overlap in Central Europe and the Mediterranean for all three species (Figures 2–6). There was a considerable overlap between the distribution of the primary hosts and the range of *I. simplex* (Figure 4) and *I. vespertilionis* (Figure 5). *Ixodes ariadnae* shows the smallest range (Figure 3), followed by *I. simplex* (Figure 4) and *I. vespertilionis* (Figure 5). Two of the three species also occurred south of the Mediterranean Sea, in Africa, though all records of *I. ariadnae* lay in Central Europe and the Middle East (Anatolia). Most host-collected ixodid ticks came from bats caught close to underground roosts (90.2%), regardless of whether the hosts were cave-dwellers (91.3%, *n* = 2,367) or crevice dwellers (79.3%, *n* = 517). Records of hard ticks on crevice dwellers were made mainly in the autumn (71% of all records in August–October). A single tick species, *I. ariadnae*, showed strong seasonality, with 93.4% of records occurring from August to October. Adult females of *I. simplex* also showed clumped seasonal occurrence, with over 62% collected in spring (April–June), although only 22% of tick-infested hosts were recorded in spring. We found no marked seasonal differences in the distribution of *I. vespertilionis* collected from hosts, though slightly more records came from spring. Two bat-specialist tick species were found on humans (*I. simplex* and *I. vespertilionis*, each in a single instance). Both species were also collected from dogs, and *I. vespertilionis* was found on horses and wild boars.

**Figure 7:**
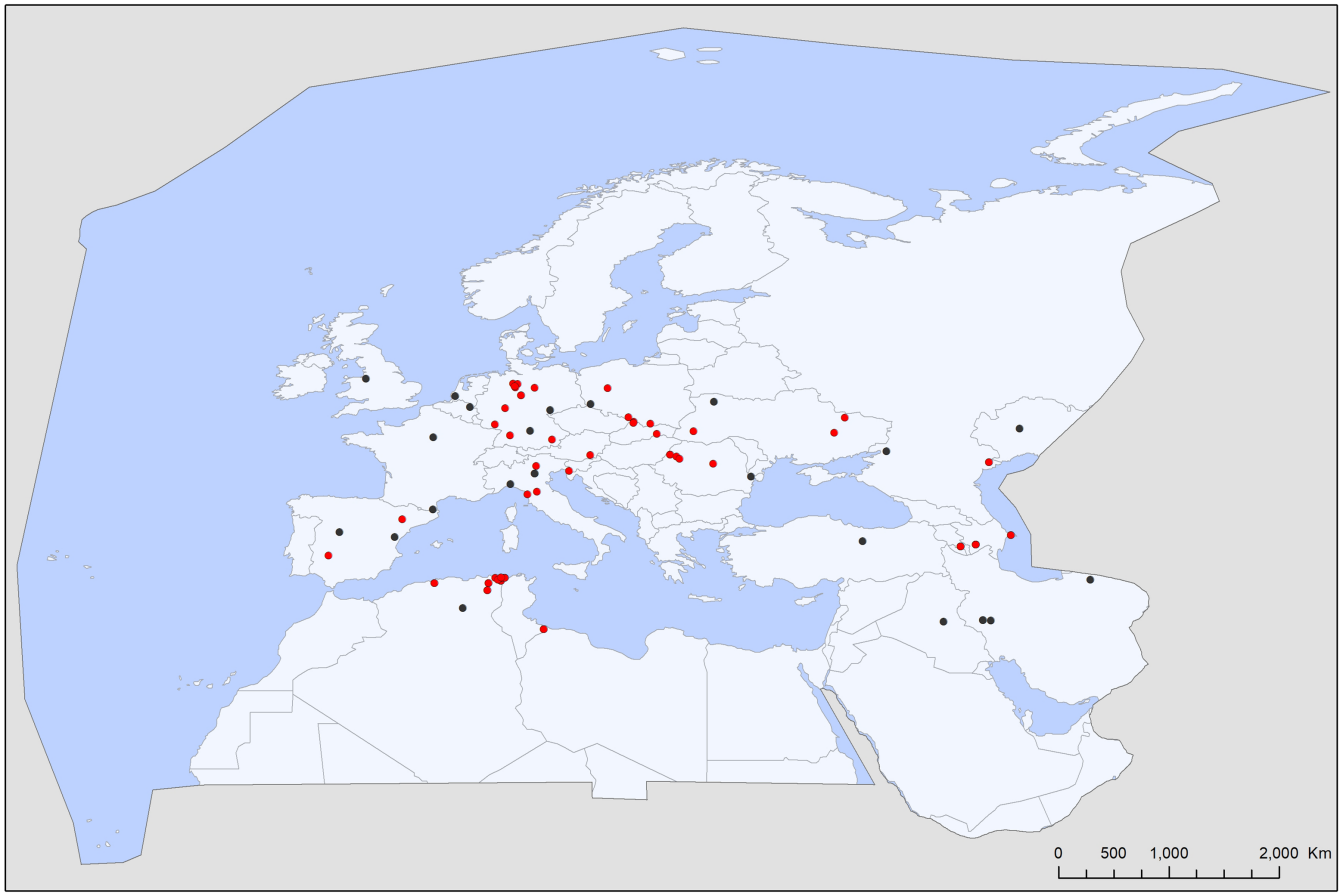
Map with the geographic distribution of other tick species (non-bat ticks) recorded on bats in the Western Palearctic.

This survey recorded 110 host–parasite associations involving 27 bat species and other tick species (18 species, 149 individuals; Table 3). Most of these records involved generalist ticks, e.g., *Ixodes ricinus* (61 cases, 90 individuals, 60.4% of non-specialist tick records) and *Rhipicephalus sanguineus s.l.* (31 cases, 46 individuals, 30.8% of non-specialist tick records; Figures 6, 7). Even bird-specialists (*I. arboricola*, *I. lividus*) or rodent-specialists (*I. redikorzevi*, *I. trinaguliceps*) were occasionally recorded. The geographic range of generalist tick records showed a primarily southern distribution, with most being collected in the western part of the Mediterranean region (Figure 7). Wide-range, generalist ticks (*I. ricinus* and *R. sanguineus s.l.*) were mostly found on larger, heavier bat species (mean body weight for these hosts was 16.75 g vs. 15.16 g for the rest of generalist tick’s host). These ticks were evenly distributed all over the region (Figure 7, red dots), on both crevice-and cave-roosting species, with *Pipistrellus pipistrellus* hosting the most tick species (7 tick species), while most ticks were collected from *M. myotis* (16 cases) and *M. schreibersii* (11 records).

**Table 3:** List of other (generalist or bird specialist) tick species recorded on bats, with bat host species and number of occurrences.

Tick species	Host species	Number of cases
*Dermacentor marginatus*	*Myotis blythii*	1
	*Pipistrellus pipistrellus*	1
	*Plecotus macrobullaris*	1
	*Rhinolophus mehelyi*	2
*Dermacentor reticulatus*	*Plecotus auritus*	1
*Haemaphysalis concinna*	*Pipistrellus pipistrellus*	1
*Haemaphysalis erinacei*	*Nyctalus noctula*	2
	*Otonycteris hemprichii*	1
	*Pipistrellus pipistrellus*	1
*Haemaphysalis punctata*	*Plecotus auritus*	1
	*Rhinolophus ferrumequinum*	2
*Haemaphysalis sulcata*	*Eptesicus ognevi*	1
	*Otonycteris hemprichii*	1
*Haemophysalis concinna*	*Chiroptera* spp.	1
	*Myotis mystacinus*	1
*Hyalomma dromedari*	*Pipistrellus kuhlii*	1
*Ixodes arboricola*	*Nyctalus noctula*	1
	*Pipistrellus nathusii*	1
*Ixodes eldaricus*	*Rhinolophus mehelyi*	1
*Ixodes hexagonus*	*Myotis myotis*	1
	*Rhinolophus ferrumequinum*	3
*Ixodes lividus*	*Pipistrellus pipistrellus*	2
*Ixodes redikorzevi*	*Pipistrellus pipistrellus*	1
	*Rhinolophus mehelyi*	1
*Ixodes ricinus*	*Eptesicus isabellinus*	1
	*Eptesicus serotinus*	1
	*Myotis bechsteinii*	1
	*Myotis blythii*	1
	*Myotis daubentonii*	2
	*Myotis myotis*	13
	*Myotis mystacinus*	1
	*Myotis nattereri*	1
	*Nyctalus noctula*	4
	*Pipistrellus kuhlii*	1
	*Pipistrellus pipistrellus*	1
	*Plecotus auritus*	1
	*Rhinolophus euryale*	2
	*Rhinolophus ferrumequinum*	2
	*Rhinolophus hipposideros*	6
	*Rhinolophus mehelyi*	2
	*Tadarida teniotis*	2
	*Vespertilio murinus*	1
*Ixodes trianguliceps*	*Myotis myotis*	1
	*Nyctalus noctula*	1
*Rhipicephalus rossicus*	*Eptesicus serotinus*	2
	*Pipistrellus pipistrellus*	3
*Rhipicephalus sanguineus*	*Eptesicus isabellinus*	2
	*Hypsugo savii*	2
	*Miniopterus schreibersii*	3
	*Myotis capaccinii*	2
	*Myotis punicus*	8
	*Rhinolophus hipposideros*	2
*Rhipicephalus turanicus*	*Miniopterus schreibersii*	8

Several viral, bacterial, and apicomplexan pathogens were identified in all three bat specialist tick species. DNA of at least eight bacteria, six piroplasmids, a haemosporidian and five viruses were identified in *I. simplex*, with similar number of bacteria, but less diverse apicomplexan and virus presence in *I. vespertilionis*. The least studied species (*I. ariadnae*, four studies), harbored DNA of two bacteria and a single piroplasmid (Table 4).

**Table 4:** DNA of pathogens detected in bat specialist ticks of the Western Palearctic bats.

Tick	Pathogen group	Pathogen species	Reference
*Ixodes ariadnae*	Bacteria	*Bartonella* sp.	Hornok et al. (43) and McKee et al. (31)
		*Wolbachia* sp.	Szentiványi et al. (45)
	Piroplasmida	*Babesia vesperuginis*	Hornok et al. (17)
*Ixodes simplex*	Bacteria	*Mycoplasma* spp.	Hornok et al. (16), Corduneanu et al. (11) and Wang et al. (51)
		*Anaplasma phagocytophilum*	Hornok et al. (16)
		*Anaplasma ovis*	Moraga-Fernández et al. (18)
		*Bartonella* spp.	Hornok et al. (16)
		*Rickettsia slovaca*	Moraga-Fernández et al. (18)
		*Rickettsia aeschlimanii*	Moraga-Fernández et al. (18)
		*Coxiella burnetii*	Moraga-Fernández et al. (18)
		*Occidentia massiliensis*	Moraga-Fernández et al. (18)
		*Neoehrlichia mikurensis*	Szentiványi et al. (45)
	Piroplasmida	*Babesia crassa*	Hornok et al. (17)
		*Babesia venatorum-like*	Hornok et al. (17)
		*Babesia canis*	Hornok et al. (17)
		*Theileria capreoli*	Hornok et al. (17)
		*Theileria orientalis*	Hornok et al. (17)
		*Theileria* sp. OT3	Hornok et al. (17)
	Haemosporida	*Polycromophilus melanipherus*	Sándor et al. (30)
	Virus	Jingmen tick virus	Dincer et al. (52)
		Lloviu virus	Kemenesi et al. (53)
		*Flavivirus*	Moraga-Fernández et al. (18)
		Crimean Congo Hemoragic Fever virus	Moraga-Fernández et al. (18)
		*Nairovirus*	Moraga-Fernández et al. (18)
		*Orthonairovirus*	Moraga-Fernández et al. (18)
*Ixodes vespertilionis*	Bacteria	*Bartonella* sp.	Hornok et al. (16, 43) and Szentiványi et al. (45)
			Hornok et al. (16)
			Szentiványi et al. (45)
		*Bartonella tamiae*	Leulmi et al. (44)
		*Wolbachia* sp.	Hornok et al. (43)
			Tian et al. (46)
		*Rickettsia* sp.	Tian et al. (46)
		*Rickettsia africae*	Tian et al. (46)
		*Coxiella burnettii*	Leulmi et al. (44)
		*Coxiella* sp.	Tian et al. (46)
		*Neoehrlichia mikurensis*	Szentiványi et al. (45)
		*Midichloria* sp.	Cafiso et al. (42)
	Haemosporida	*Polycromophilus murinus*	Sándor et al. (30)
	Piroplasmida	*Babesia vesperuginis*	Hornok et al. (17)
		*Babesia crassa*	Hornok et al. (17)
	Virus	Iflavirus IvespIV	Daveu et al. (48)
		Issyk-Kul virus	L’vov et al. (49)

## Discussion

Our survey identified three ixodid ticks specialized on bats in the Western Palearctic, all of which belong to the genus *Ixodes*. These ticks were recorded from 31 bat species in the region (approximately 40% of all regularly occurring bats; Table 2), with hosts belonging to several bat families, including Hipposideridae, Miniopteridae, Molossidae, Rhinolophidae, Rhinopomatidae, and Vespertilionidae (37). The ticks primarily target cave-dwelling bat species (>90% of tick records with known hosts; see Table 2; Supplementary Table S1) but were also collected from crevice-dwellers during the swarming or hibernation periods when these bats regularly use underground habitats (37). Records of free stages for all three species were exclusively made underground, either inside active bat roosts in caves and mines (98.5%) or in large buildings and cellars with similar environmental conditions, often used by the same bat species. This co-occurrence is likely a result of shared evolutionary history or ecological limiting factors. *Ixodes ariadnae, I. simplex* and *I. vespertilionis* are close relatives, all three belong to the morphologically well documented *Pholeoixodes* subgenus, and their divergence supposedly happened only after a host shift of their common ancestor, likely originating from birds (38, 39). Ecological factors related to the hosts may also contribute to this sympatric occurrence, limiting tick-host interactions to specific spatial environments. All but one cave-dwelling bat species in the Western Palearctic are insectivorous (the fruit-eating *Rousettus aegyptiacus* is the exception, though no ixodid tick has been recorded from this bat). These bats spend most of their time in active flight away from roosts, spatially limiting the opportunity for ticks to access potential hosts to the interiors of the underground roosts.

Bat specialist ixodid ticks show wide distribution, two species occurring all over Europe, North Africa and the Middle East, however, the recently described *I. ariadnae* (21) was not yet found in Africa (Figure 3). The ranges of all three species overlap in Central and Eastern Europe and the Middle East, while only *I. vespertilionis* is found at northern latitudes, and *I. ariadnae* was not reported from most Mediterranean regions. There is significant overlap in the host spectrum of *I. ariadnae* and *I. vespertilionis*, with *Myotis myotis* serving as an important host for both species (Figure 1). The overlap with the hosts of *I. simplex* is less pronounced (Table 5) due to the strict host specificity of this species, which primarily parasitizes *M. schreibersii* (40, 41). Two tick species show distributions that extend well beyond the range of their primary bat host (Figures 4, 5), however, the range of *I. ariadnae* is far reduced in comparison to its primary hosts’ range, with documented records laying only in the central part of the overlapping range of its primary hosts (Figure 3). We suggest that this may be caused by several factors, like potential misidentification (for example the critical evaluation of samples collected and formerly identified as *I. vespertilionis* may complete this picture) and by reduced sampling effort in the main occurrence season (the species shows high seasonality in occurrence, limiting the chances of on-host capture, see also (40)).

**Table 5:** List of bat species (Chiroptera) and their role as primary and non-primary bat-specialist hard tick (Ixodidae) hosts in the Western Palearctic (N—number of hosts with ticks).

Bat species	N	Primary tick species	Non-primary tick species
*Asellia tridens*	2	*–*	*Ixodes vespertilionis*
*Barbastella barbastellus*	7	*–*	*Ixodes ariadnae, Ixodes vespertilionis*
*Eptesicus serotinus*	8	*–*	*Ixodes vespertilionis*
*Miniopterus schreibersii*	1,507	*Ixodes simplex*	*Ixodes vespertilionis*
*Myotis alcathoe*	13	*Ixodes ariadnae*	*Ixodes ariadnae, Ixodes vespertilionis*
*Myotis bechsteinii*	35	*Ixodes ariadnae*	*Ixodes ariadnae, Ixodes vespertilionis*
*Myotis blythii*	54	*–*	*Ixodes ariadnae, Ixodes simplex, Ixodes vespertilionis*
*Myotis brandtii*	6	*–*	*Ixodes ariadnae, Ixodes vespertilionis*
*Myotis capaccinii*	19		*Ixodes vespertilionis*
*Myotis dasycneme*	8	*–*	*Ixodes ariadnae, Ixodes vespertilionis*
*Myotis daubentonii*	86	*Ixodes ariadnae*	*Ixodes simplex, Ixodes vespertilionis*
*Myotis emarginatus*	68	*Ixodes ariadnae*	*Ixodes simplex, Ixodes vespertilionis*
*Myotis myotis*	195	*Ixodes ariadnae, Ixodes vespertilionis*	*Ixodes simplex*
*Myotis mystacinus*	44	*–*	*Ixodes vespertilionis*
*Myotis nattereri*	37	*–*	*Ixodes ariadnae, Ixodes simplex, Ixodes vespertilionis*
*Myotis punicus*	186	*Ixodes vespertilionis*	*–*
*Nyctalus leisleri*	2	*–*	*Ixodes simplex, Ixodes vespertilionis*
*Nyctalus noctula*	2	*–*	*Ixodes vespertilionis*
*Pipistrellus kuhlii*	5	*–*	*Ixodes simplex, Ixodes vespertilionis*
*Pipistrellus nathusii*	2	*–*	*Ixodes vespertilionis*
*Pipistrellus pipistrellus*	6	*–*	*Ixodes vespertilionis*
*Pipistrellus pygmaeus*	2	*–*	*Ixodes ariadnae, Ixodes vespertilionis*
*Plecotus auritus*	17	*Ixodes ariadnae*	*Ixodes vespertilionis*
*Plecotus austriacus*	2	*–*	*Ixodes vespertilionis*
*Rhinolophus blasii*	10	*–*	*Ixodes vespertilionis*
*Rhinolophus euryale*	95	*Ixodes vespertilionis*	*Ixodes simplex*
*Rhinolophus ferrumequinum*	671	*Ixodes vespertilionis*	*Ixodes ariadnae, Ixodes simplex*
*Rhinolophus hipposideros*	463	*Ixodes vespertilionis*	*Ixodes ariadnae, Ixodes simplex*
*Rhinolophus mehelyi*	24	*–*	*Ixodes ariadnae, Ixodes simplex*
*Rhinopoma muscatellum*	1	*–*	*Ixodes vespertilionis*
*Vespertilio murinus*	1	*–*	*Ixodes vespertilionis*

The geographical distribution of *I. ariadnae* and *I. vespertilionis* only partially overlaps with the distribution of their main hosts (Figures 3, 5). This discrepancy is likely due to other limiting factors beyond host range, such as climatic conditions, which may differ at the southern and northern borders of their ranges. However, the presence of bat-specialist ticks is likely not directly limited by climate, as these ticks are primarily found off-host inside underground roosts with optimal climatic conditions. This pattern is clearly visible in Figure 2, where tick distribution is plotted against limestone bedrock, which hosts more than 91% of tick occurrences due to the presence of karst formations (caves).

*Ixodes vespertilionis* has the largest distribution range, extending from Britain in the west to the Urals in the east and covering North Africa and the Middle East (Figure 5). The easternmost limit likely extends beyond the borders of the Western Palearctic. However, recent assessments of *I. vespertilionis* specimens from the Eastern Palearctic and Oriental regions revealed several new species (23, 24). This species is primarily associated with horseshoe bats (*Rhinolophus* spp.) but is also a common parasite of the three large *Myotis species* (*M. blythii, M. myotis*, and *M. punicus*). It is also scarcely recorded on other vesper bats which frequent caves (Table 5 and Figure 1), fact which may help to interpret its occurrences far from the main hosts’ range (Figure 5). The species accounts for the bulk of unengorged tick records collected in caves, due to its habit of questing on cave walls (40). The highly biased sex ratio of free stages noted in this species may be explained by males not feeding and potentially living longer than females, which die after egg laying (hence more chances of encounter on roost walls). There is a slight seasonality in the occurrence of adult free stages, with more records noted during summer months, though this may be due to more frequent cave visits during this period rather than actual seasonality of the species. Several studies detected DNA of pathogenic bacteria (16, 42–46), piroplasms (17, 47) and viruses (48, 49) in *I. vespertilionis* individuals, both in host collected and free ticks (Table 4). While definitive proof of a vectorial role of this tick species for these pathogens is lacking, its wide distribution, diverse host range, and ubiquitous presence in most bat shelters suggest a significant potential role in pathogen transmission. Moreover, a recent study performing blood-meal analyses managed to detect DNA of non-bat provenience in several adult tick individuals, thus highlighting the chances for pathogen transfer between wide range of host species (31 known species of bat hosts) and other mammals (e.g., dogs, horse and wild boar, (45)), or humans (19).

*Ixodes simplex* is a nest-dwelling tick, highly gregarious by nature, staying hidden in crevices near its main host colonies (*M. schreibersii*) (40). It is strictly host-specific, being parasitic almost exclusively on *M. schreibersii* and rarely found on other bat species (<1.5% of occurrences collected from 13 different bat species, mainly cave-dwellers roosting in sympatry with *M. schreibersii*). The geographic distribution strongly overlaps with the main distribution of its host, showing a strong mutual relationship with this bat species. Northern outlier records were reported from areas where its host was present in the past (50), while records in the Middle East mostly represent observations on its sister species, the pale bent-winged bat (*Miniopterus pallidus*). It is common on its hosts, occurring in every roost regularly used by *M. schreibersii*, showing a constant presence and likely influencing the spatial organization of these bats (41). This tick shows high seasonality in its on-host occurrences, with the highest prevalence and intensity recorded in spring/early summer, sometimes causing detrimental effects on specific host individuals (20). While *I. simplex* is suspected to vector several bacterial (11, 16, 18, 45, 51), parasitic (17, 47), and viral pathogens (18, 52, 53), there is no unequivocal proof for these roles.

*Ixodes ariadnae* was recently described from Central European bats (21, 38) and remains a rare bat ectoparasite, with most records geographically limited to a narrow east–west belt between 44° and 51°N latitude, primarily in Europe. Compared to the distribution range of its primary hosts, *I. ariadnae* shows a highly reduced distribution area. We suggest that this range reflects the spatial extent of recent bat-tick studies rather than the actual distribution, which is expected to increase with future research efforts. This species displays strong seasonality, with 92.2% of host-collected ticks recorded during August–September, coinciding with the autumn swarming of bats (45). While there are fewer than 100 records of *I. ariadnae*, it has a relatively diverse host range, with 15 known bat hosts (Figure 1; Tables 1, 2). Most hosts (*n* = 10, 67%) are crevice-dwelling forest bats, which only use underground roosts during swarming or hibernation. Questing adults of *I. ariadnae* were mainly collected during winter months, though this is likely due to limited access to cave sections occupied by this species (S. Hornok, pers. comm.) rather than true seasonal activity peaks. Only a handful of studies have recorded pathogens in *I. ariadnae* (Table 4), detecting DNA from bacteria (16, 31, 45) and piroplasms (17).

All, but one Western Palearctic bat species are insectivorous and most species are hunting during flight, relying mainly on insects in flight. In consequence, ticks not using caves or other bat roosts rarely gain access to bat hosts. Thus, presence of generalist ticks on bats is a rare phenomenon, with <1.7% of all tick encounters related to bats represent other species than the three bat-specialist *Ixodes*. Truly generalist ticks (*I. ricinus, R. sanguineus* s.l.) made up the bulk of these records and these mainly targeted large-bodied species regularly hunting on the ground (*M. blythii, M. myotis*, and *M. punicus*). Other tick species are rarely recorded on bats and are mostly accidentals. Some of these ticks are bird-specialist nest-dwellers, e.g., *I. arboricola* (regular in tree crevices and bird nest boxes) or *I. lividus* (a tick species using nest-holes dug by sand martins, *Riparia riparia* (54)), species which may get access to bats roosting in these bird-nests. Other species are ticks associated to carnivora, which regularly occur in caves (*Haemaphysalis erinacei* and *I. hexagonus* (55)).

Bats are frequently parasitized by ticks, and these ticks can host pathogenic bacteria, parasites, or viruses. Certain bat species may act as bridging hosts, carrying not only bat-specialist ticks but also generalist ticks, thus they may have a particular importance from One Health perspective (56). Additionally, a recent study detected high levels of non-bat host DNA in free-living bat ticks, further highlighting the potential for bridging bat-related pathogens to other hosts.

## Data Availability

The original contributions presented in the study are included in the article/Supplementary material, further inquiries can be directed to the corresponding author.

## Footnotes

^1^ www.gbif.org

^2^ https://spatial.nbnatlas.org/
